# No association between body dissatisfaction and attentional bias towards lower-weight bodies in a non-clinical sample of women: an eye-tracking study

**DOI:** 10.1098/rsos.252073

**Published:** 2026-10-28

**Authors:** Charlotte Crisp, Yufei Jin, Mary-Kate S Nealon, Helen Low, Jon Heron, Iain D. Gilchrist, Ian Penton-Voak, Helen Bould

**Affiliations:** ^1^ Population Health Sciences, Bristol Medical School, University of Bristol, Bristol, UK; ^2^ NIHR Bristol Biomedical Research Centre, University of Bristol, Bristol, UK; ^3^ School of Psychology and Neuroscience, University of Bristol, Bristol, UK; ^4^ Centre for Academic Mental Health, Population Health Sciences, University of Bristol, Bristol, UK; ^5^ Gloucestershire Health and Care NHS Foundation Trust, Brockworth, UK

**Keywords:** body dissatisfaction, attentional bias, eye tracking, fixation duration, women, partial replication, pre-registered

## Abstract

Body dissatisfaction is a key risk factor for psychiatric illnesses and is a modifiable target. Previous gaze-tracking research suggests that body dissatisfaction is related to greater attentional bias towards lower-weight bodies. Here, we aimed to partially replicate and extend previous work to verify this association by using realistic body stimuli and a validated scale of body dissatisfaction. Sixty, mostly young, women from the general population underwent eye-tracking while viewing picture pairs of women's bodies morphed to lower and higher weights. Attentional bias was measured as percentage fixation duration, fixation count and first fixations on lower-weight compared to higher-weight bodies. There was weak evidence that all women fixated for longer on lower-weight bodies, and that participants with higher body dissatisfaction spent less time fixating on lower-weight bodies, contrary to our hypotheses and to previous findings, but this effect was not robust to adjustment for age and body mass index. This lack of replication suggests this association is not robust in non-clinical populations. Instead, an overall bias towards lower-weight bodies could contribute to higher levels of body dissatisfaction and risk of mental health problems in younger women. Future studies measuring attentional biases in realistic settings and in clinical populations with eating disorder symptoms are needed.

## 1. Introduction

Body dissatisfaction refers to negative thoughts and emotions regarding one's body, which is thought to stem from a perceived difference between one's own body and an ‘ideal’ body [1]. Body dissatisfaction is a key risk factor for psychiatric illnesses such as eating disorders (e.g. anorexia nervosa, bulimia nervosa) and depression and is also associated with the maintenance of these illnesses [2–4]. More broadly, body dissatisfaction is associated with health-compromising behaviours such as smoking, drinking, drug use and inappropriate exercise (compulsive or inadequate physical activity) [5–7]. Body dissatisfaction is therefore an important, potentially modifiable target for the prevention of mental and physical health problems at a public health level. An improved mechanistic understanding of body dissatisfaction is needed in order to develop new approaches to reduce it.

One conceptualization of body dissatisfaction in the literature relates to comparison with the ‘thin ideal'. Our previous work has shown that repeated exposure to lower-weight (versus higher-weight) bodies leads to the subsequent perception of others' bodies as larger, and reduces satisfaction with own body [8]. Such exposure also shifts perception of ‘normal’ and ‘ideal’ body size, such that both appear lower-weight following exposure to images of lower-weight bodies [9]. Cognitive-behavioural theories suggest that body dissatisfaction may cause preferential attention towards body-related stimuli [10], and our recent meta-analysis suggested that high body dissatisfaction was associated with greater attention towards lower-weight (versus higher-weight) bodies, as measured by gaze tracking [11]. Such an attentional bias may, in turn, further exacerbate body dissatisfaction via a negative feedback loop. Attentional biases towards lower-weight bodies could therefore be a target for cognitive retraining interventions. For example, attention and cognitive bias modification interventions that train individuals to orient away from disorder-relevant stimuli (e.g. lower-weight bodies) may be helpful for reducing body dissatisfaction and risk for eating disorders. Similar interventions have been used for other mental health conditions, e.g. depression [12].

The gaze-tracking studies included in the previous meta-analysis had varied methodology. For example, some studies used unrealistic stimuli (e.g. digitally constructed three-dimensional images of bodies [13]) or non-body-related images as control stimuli (e.g. gardening-related items [14]). There was also some variation in whether an attentional bias was demonstrated, and the evidence for the pooled gaze-tracking effect may have been inflated owing to publication bias. The meta-analysis also highlighted inconsistencies in the way that body dissatisfaction is measured, including questionnaires focused on body appreciation, body image, body shape, body weight or ‘fatness’ and (dis)satisfaction. One key study by Stephen *et al*. [15], which found evidence of an attentional bias, used a free-viewing task to examine the relationship between body dissatisfaction (measured on a single-item body dissatisfaction measure) and the number of fixations and fixation duration on lower-weight bodies compared with higher-weight bodies [15]. However, a validated measure of body dissatisfaction (beyond the single-item measure used by Stephen and colleagues) is needed to further corroborate the findings and lay the groundwork for developing interventions.

The aim of the current study, therefore, was to partially replicate and extend the work by Stephen *et al*. [15] to verify the association between body dissatisfaction and attention towards lower-weight bodies. We employed a similar free viewing task with eye-tracking. We pre-registered our planned analyses, used a realistic set of body stimuli and used a validated measure of body dissatisfaction: the body dissatisfaction subscale of the Eating Disorder Inventory 3 (EDI-3). This scale assesses dissatisfaction with body shape and size (a central feature of the psychopathology of many eating disorders), and is widely used in clinical and research settings, has strong theoretical grounding, and good reliability and validity. We also collected information on participant weight and height, which can influence body dissatisfaction. We restricted our sample to women, owing to higher levels of body dissatisfaction, and prevalence of eating disorders in this population compared to men [16,17], and also to more closely replicate the work by Stephen *et al*. [15].

### 1.1. Study objectives and hypotheses

Our objective was to use eye-tracking to investigate the relationship between body dissatisfaction and attention towards lower-weight bodies in a sample of adult women. We hypothesized that higher body dissatisfaction would be associated with an attentional bias towards lower-weight bodies when compared to higher-weight bodies, as measured by (i) a greater percentage of time fixating on lower-weight bodies (fixation duration), and (ii) a greater percentage of fixations on lower-weight bodies (fixation count). In an exploratory hypothesis, body dissatisfaction was also predicted to be associated with initial attentional capture by lower-weight bodies, indexed by the proportion of first fixations on lower-weight bodies.

## 2. Material and methods

### 2.1. Study design

This was an observational study of women performing a free-viewing eye-tracking task. Participants attended a single in-person session at the School of Psychological Science, University of Bristol (UK) between June and October 2024. We pre-registered the study protocol on the Open Science Framework (OSF): https://doi.org/10.17605/OSF.IO/C8YX9. Methods and results have been written according to the minimum reporting standards for eye-tracking studies [18].

### 2.2. Participants and recruitment

We recruited participants via posters in the local Bristol area and online (e.g. University mailing lists). Participants were included in the study if they identified as a woman, were aged 18 or over, had English language fluency and normal or corrected-to-normal vision. Participants were excluded from participating if they self-reported a past or present diagnosis of an eating disorder or if they wore glasses and were unable to wear contact lenses to correct their vision (to avoid less reliable eye tracking through glasses). Participants who completed the study were reimbursed with a £5 Amazon voucher.

### 2.3. Sample size determination

A previous study [15] showed that body dissatisfaction was associated with increased attention (measured using percentage fixation duration) towards lower-weight bodies (beta = −3.09, Fisher's *Z* = 0.39). Assuming this (medium) effect size, we calculated that we would need a sample size of *n* = 52 to detect an effect with 80% power and alpha = 0.05, using linear regression with body dissatisfaction score as the exposure and fixation duration to lower-weight bodies as the outcome measure. We aimed to recruit 60 participants to account for loss of data owing to eye-tracking calibration issues.

### 2.4. Questionnaire measures

We collected demographic information on age, gender, sex assigned at birth, weight and height at the start of the study. We measured body dissatisfaction using the body dissatisfaction subscale of the EDI-3 [19]. This is a 9-item measure of body dissatisfaction focusing on size and shape characteristics. It uses items such as ‘I think my hips are too big’ and asks respondents the degree to which they agree with the statements on a scale of 0 (never), 1 (rarely), 2 (sometimes), 3 (many times) or 4 (always). Total scores range from 0 to 36, where higher scores represent higher body dissatisfaction. Baseline anxiety symptoms were assessed using the Generalised Anxiety Disorder Questionnaire (GAD-7) [20], which was included to reduce the likelihood of participants guessing the primary purpose of the questionnaires (measuring body dissatisfaction).

### 2.5. Eye-tracking task

The task was presented using Eyelink software, which also controlled the collection of the eye movement data (SR Research Ltd, version 1.11.900). Eye movements were recorded using a video-based eye-tracker (Eyelink 1000 Plus) providing gaze co-ordinate data at a sample rate of 1000 Hz. The eye tracker recorded horizontal and vertical gaze positions in pixels on the screen, where (0, 0) corresponded to the top-left of the display screen. Stimuli were taken from a set of photographic images of female bodies in black crop tops and shorts, each morphed between low and high weight using image morphing techniques to produce nine image versions of each original body [21]. In this study, we selected the six bodies that had both lower-weight and higher-weight image versions that at least 50% of the participants (from Skinner *et al*. [21]) had rated as underweight and overweight, respectively. In each trial, two images were presented side by side: one lower-weight body and one higher-weight body (of the same identity; see figure 1 for example). This resulted in six image pairs. All images were cropped to the same size (measuring 281 pixels × 775 pixels). The faces in the images were blurred (for anonymity and to reduce attention towards faces). Each image pair was presented four times, in a random order, and the side of the screen on which each body appeared was counterbalanced. Pre-defined areas of interest (AOI) measuring 207 pixels × 626 pixels were placed on the images around the outside of the lower-weight and higher-weight bodies but excluding their heads (figure 1). Neutral images were also presented to break up the viewing of images of bodies; these trials were not analysed. Twelve images of puppies taken from Google Images were made into six image pairs and randomly interspersed between images of bodies trials, as well as being presented for the final three trials of the task as a ‘washout’ period. Participants viewed a total of 36 trials (six pairs of bodies, each presented four times, and 12 images of puppies). Each image pair was presented for 6 seconds, and an automatic drift check was performed between each trial. The whole eye-tracking task took about 3 minutes to complete.

**Figure 1.**
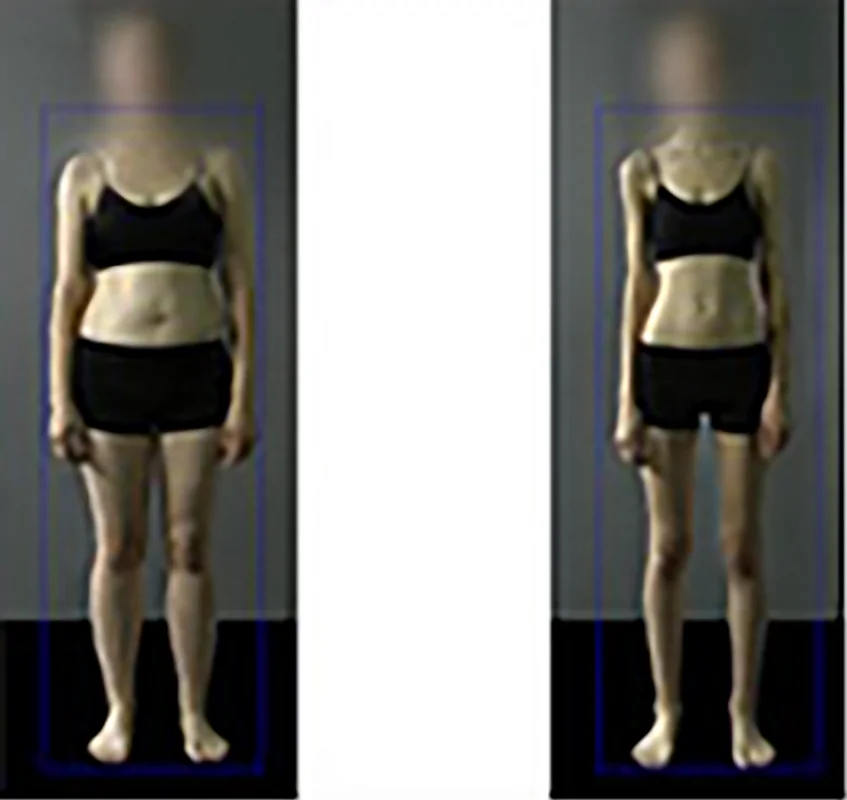
An example image pair for test trials. A higher-weight body is presented next to a lower-weight body of the same female identity. The boxes outlined in blue on the images show the pre-defined areas of interest.

### 2.6. Procedure

Participants were asked to attend the eye-tracking laboratory wearing contact lenses (if needed) and without heavy eye-make up (to aid eye-tracking). Participants gave informed consent and filled out the questionnaires described above on Qualtrics. The researcher determined the participant's dominant eye (which was then tracked during the task) using a Miles test [22]. In this test, participants are asked to bring both hands placed in a diamond shape together up to one of their eyes to view the researcher—this eye was then considered the dominant eye. Participants placed their chin on the chinrest and their forehead against the forehead rest and adjusted the height of the chair. The chin rest was set up approximately 57 cm away from the display screen, and so the participant's eye line was level with the middle of the screen. The eye-tracking camera was placed at the bottom centre of the display screen. The lights were turned off, and participants were asked to keep their heads as still as possible throughout the task.

Camera thresholding was performed on the Eyelink computer screen using auto-threshold or by manually adjusting so that only the pupil appeared ‘blue’. Pupil-only tracking mode was used. Next, participants underwent the built-in Eyelink 9-point calibration procedure by viewing a moving black dot around a 3 × 3 grid pattern until the researcher determined the eye movements were stable and replicated the grid pattern. In order to confirm calibration, this was followed by a 9-point validation procedure using the same grid pattern. Calibration and validation procedures were performed until the mean error score was < 0.4° (meaning 4 mm spatial precision). The researcher then left the room (to reduce the possibility of desirability bias), and the task instructions were presented on the screen for 10 seconds. Participants were instructed to view the images presented as naturally as possible and were not asked to respond in any way. Between trials, participants were asked to refocus their gaze on a black dot in the centre of the screen, and the subsequent trial was automatically presented if they had stable fixation data and fixated within five degrees of the dot (automatic drift check). If the drift check failed during the task, single-point validation was performed again, and the task automatically continued from the point at which it had stopped. If validation was unsuccessful, full calibration and validation were performed again before continuing the task. At the end of the experiment, participants were debriefed and reimbursed. The whole study session took up to 30 minutes.

### 2.7. Ethical considerations and informed consent

Ethics approval was obtained from the School of Psychological Science Research Ethics Committee at the University of Bristol (approval code: 19187). The study was conducted according to the revised Declaration of Helsinki (2013) and the 1996 ICH Guidelines for Good Clinical Practice E6(R2).

### 2.8. Statistical methods

#### 2.8.1. Quality control

Only participants with precise measures of gaze position before starting the task (defined as an average validation error score < 0.4°) were included in analysis. Participants who failed to complete all trials, failed the drift check more than three times during the task or spent more than 40% of the task fixating outside the pre-defined AOIs (see below) were excluded from analyses.

#### 2.8.2. Statistical analysis

Analyses were performed on test trials. The primary outcome measure was the percentage of fixation duration on lower-weight bodies (calculated as total fixation duration within the lower-weight body AOI divided by total presentation time of stimuli (6 seconds)). The secondary outcome measure was the percentage of fixations on lower-weight bodies (total number of fixations within the lower-weight body AOI divided by the total number of fixations). Linear regression models were used to test whether the body dissatisfaction score was associated with the primary and secondary outcome measures. A linear regression analysis was also performed, using the percentage of first fixations towards lower-weight bodies (total number of first fixations on lower-weight bodies divided by total number of trials with valid first fixations) as the outcome measure. This was used as a measure of initial attention capture to better understand attentional bias. The first fixation can be considered a measure of the earliest allocation of attention, often outside conscious control, driven by the bottom-up saliency of the stimulus and before deeper processing of the stimulus occurs (reflected by the total fixation duration) [23]. A trial was considered to have valid first fixations if: (i) first fixations on at least one of the AOIs occurred between 0 and 6000 ms from stimulus onset, and (ii) the first fixation duration on both AOIs lasted between 100 and 6000 ms. Trials that did not meet these criteria were excluded. For each participant, the number of valid first fixations on lower-weight bodies was then divided by the total number of valid trials to yield the final percentage. Models are presented unadjusted and adjusted for age and body mass index (BMI).

We also conducted exploratory analyses that were not specified in our pre-registered protocol. This included using the percentage of fixation duration relative to fixation duration within the AOIs (rather than within the full 6 second trial). This gives a complementary measure (to the primary outcome measure) that specifically focuses on body-related attentional bias. We also ran sensitivity analyses comparing participants with the highest and lowest quartiles of body dissatisfaction scores on their measures of attention, to allow direct comparison with other previous studies (e.g. [13]), and using the single item ‘I feel satisfied with the shape of my body’ to directly compare with the analysis by Stephen *et al*. [15].

## 3. Results

### 3.1. Quality control

We recruited 60 participants. Three participants were excluded from analysis: one owing to failure to successfully calibrate the eye-tracker, one with poor gaze position precision (validation mean error score > 0.4), and one for whom more than 40% of fixations were outside the pre-defined AOIs. The final sample size was therefore *n* = 57. On average, 4.21 (s.d. = 2.68) first fixation trials per participant were considered invalid owing to first fixations on both AOIs occurring outside the range 0–6000 ms and/or first fixation duration on at least one of the AOIs lasted outside the range 100–6000 ms and were excluded from this analysis, corresponding to 17.5% of the total 24 test trials per participant.

### 3.2. Measurement uncertainty

The mean precision of gaze position across participants was 0.27° (s.d. = 0.07°). There were 18 participants (32%) who failed the auto drift check during the task (unstable eye fixation or fixation more than 5° away from the centre dot between trials) and resumed the task after single-point re-validation.

### 3.3. Participant characteristics

Participant characteristics are presented in table 1. Participants were aged between 18 and 75 years old (*M* = 27.4 years, s.d. = 9.24), and their mean BMI was 23.7 (s.d. = 3.70). Body dissatisfaction scores were within national norms (*M* = 14.2, s.d. = 6.96) [24], and participants reported mild anxiety (*M* = 5.81, s.d. = 3.61).

**Table 1.** Descriptive statistics of participant characteristics. (s.d., standard deviation; BMI, body mass index; EDI-3-BD, Eating Disorder Inventory-3-Body Dissatisfaction subscale (scores range from 0 to 36); GAD-7, Generalised Anxiety Disorder-7 (scores range from 0 to 21); IQR, interquartile range.)

	mean (s.d.)	median (IQR)	minimum	maximum
**age**	27.4 (9.24)	25.0 (9.00)	18	75
**BMI**	23.7 (3.70)	23.2 (4.25)	16.0	33.3
**EDI-3-BD**	14.2 (6.96)	14.0 (8.00)	0	31
**GAD-7**	5.81 (3.61)	5.00 (5.00)	0	17

### 3.4. General attention to bodies of different sizes

The descriptive statistics for each of the primary, secondary and exploratory outcome measures are presented in table 2. We found no robust evidence that participants spent a greater percentage of time fixating on lower-weight bodies compared to higher-weight bodies (mean difference = −3.12%, 95% confidence interval (CI) [−6.73, 0.482], *p* = 0.09). We did not find convincing evidence that participants had a higher percentage of fixation counts on lower-weight compared to higher-weight bodies (mean difference = −2.62%, 95% CI [−6.00, 0.757], *p* = 0.13). We found no evidence that participants had different percentages of first fixations on lower-weight versus higher-weight bodies (mean difference = −3.08%, 95% CI [−8.57, 2.41], *p* = 0.27). It should be noted that while the size of the region of interest (ROI) was the same for each stimulus, the higher-weight bodies occupied more space within the ROI than the lower-weight bodies. This may have corresponded to slightly more fixations on higher-weight bodies, which is a limitation of the study design. This calculation was the same for every participant, and therefore, this issue did not impact the association with body dissatisfaction.

**Table 2.** Mean (s.d.) values for fixation data on higher-weight and lower-weight bodies. (Note: percentages are presented relative to trial duration (6 seconds). s.d., standard deviation; CI, confidence interval; ms, milliseconds.)

	higher weight body stimuli	lower weight body stimuli	mean difference [95% CI]	*p*-value
**fixation duration (%)**	36.6 (7.35)	39.7 (7.85)	−3.12 [−6.73,0.482]	0.09
**fixation duration (ms)**	2277 (457)	2471 (488)	−193 [−420,32.8]	0.09
**fixation count (%)**	38.7 (7.08)	41.3 (6.99)	−2.62 [−6.00,0.757]	0.13
**fixation count**	8.01 (1.82)	8.54 (1.80)	−0.529 [−1.21,0.152]	0.13
**first fixation count (%)**	48.5 (10.3)	51.5 (10.3)	−3.08 [−8.57, 2.41]	0.27
**first fixation count**	9.56 (2.24)	10.2 (2.57)	−0.667 [−1.73, 0.396]	0.21

### 3.5. Association between body dissatisfaction and attention to bodies of different sizes

There was a weak trend that body dissatisfaction was associated with the percentage fixation duration on lower-weight bodies (*β* = −0.267, 95% CI [−0.563, 0.0288], *p* = 0.08), in the opposite direction to our hypothesis; this association was no longer evident after adjustment for age and BMI (*β* = −0.0910, 95% CI [−0.410, 0.228], *p* = 0.57). We found no evidence of an association between body dissatisfaction and the percentage of fixations on lower-weight bodies, or the percentage of first fixations on lower-weight bodies, in either unadjusted or adjusted models (see table 3). To explore whether these findings were skewed by older participants in our sample, we conducted sensitivity analyses excluding three participants (aged 45, 48 and 75) falling 1.5 × outside the interquartile range. There was no evidence that body dissatisfaction was related to our primary, secondary or exploratory outcomes in this restricted sample (supplementary material, table S1).

**Table 3.** Linear regression models of the association between body dissatisfaction and outcome measures. (Note: percentages are presented relative to trial duration (6 seconds). CI, confidence interval.)

	linear regression model 1: unadjusted *β* [95% CI]	*p*-value	linear regression model 2: adjusted^a^ *β* [95% CI]	*p*-value
**fixation duration on lower weight bodies (%)**	−0.267 [−0.563, 0.0288]	0.08	−0.0910 [−0.410, 0.228]	0.57
**fixation count on lower weight bodies (%)**	−0.199 [−0.465, 0.0670]	0.14	−0.0297 [−0.313, 0.253]	0.83
**first fixation count on lower weight bodies (%)**	−0.0113 [−0.413, 0.390]	0.96	0.0597 [−0.392, 0.512]	0.79

^a^ Adjusted for age and BMI.

### 3.6. Exploratory analyses

We explored the association between body dissatisfaction and the percentage fixation duration relative to total fixation duration within the AOIs (instead of within the full 6 second trial). Similar to above, we found a suggestion that, on average, participants spent slightly more time fixating on lower-weight bodies, although evidence for this was not robust (mean difference = −3.70%, 95% CI [−8.32, 0.919], *p* = 0.11). Participants with higher body dissatisfaction spent less time fixating on lower-weight bodies, but this was not robust to adjustment for age and BMI (*β* = −0.188, 95% CI [−0.540, 0.164], *p* = 0.29).

#### 3.6.1. High versus low body dissatisfaction

To understand whether attentional biases may be apparent when comparing those with the highest (versus lowest) levels of body dissatisfaction, and to enable direct comparison with previous work (e.g. [13]), we ran sensitivity analyses comparing those individuals whose body dissatisfaction scores were in the highest and lowest quartiles. We did not find evidence that participants in the high body dissatisfaction group (*n* = 16) differed from participants in the low body dissatisfaction group (*n* = 17) in terms of fixation duration, fixation count and first fixations to lower-weight bodies compared to higher-weight bodies. Furthermore, none of the unadjusted or adjusted linear regression models showed an association between body dissatisfaction and any of the outcome measures (see the supplementary material, tables S2–S4).

#### 3.6.2. Single-item body dissatisfaction measure

 To test whether our measure of body dissatisfaction was better represented by a single ‘overall’ measure, and to enable direct comparison to Stephen *et al*. [15], we repeated our analyses using the single item ‘I feel satisfied with the shape of my body’. Results across all analyses using this single item rather than our composite measure did not meaningfully change any of our results (supplementary material, table S5).

## 4. Discussion

This study investigated the relationship between body dissatisfaction and attention towards lower-weight bodies in a non-clinical sample of, predominantly, young, adult women using eye-tracking. Overall, we found weak, but not robust, evidence for an attentional bias (indicated by longer fixation times) towards lower-weight bodies compared to higher-weight bodies among all women, regardless of their level of body dissatisfaction. There was a suggestion that participants with higher body dissatisfaction spent less time fixating on lower-weight bodies, contrary to our hypotheses and to previous findings [11,15], but this was not robust. It is possible that this relationship instead reflects attentional avoidance of lower-weight bodies as a protective mechanism. Similar avoidance patterns have been observed in anxiety disorders, where individuals may divert their attention away from salient stimuli. However, this effect was not robust to adjustment for age and BMI, and therefore, we suggest that overinterpretation should be avoided. Exploratory follow-up analyses showed that these null findings did not change when we considered attentional biases specific to body-related stimuli; compared those scoring in the highest compared to the lowest quartiles for body dissatisfaction; or used a single-item measure of overall body dissatisfaction. Therefore, this study did not replicate previous findings from [15], and did not find evidence of an attentional bias to smaller bodies among women with higher levels of body dissatisfaction.

Our recent meta-analysis examining the relationship between high body dissatisfaction and attentional biases towards lower-weight bodies highlighted that support for this relationship was highly influenced by how attentional biases were measured. Studies using dot-probe tasks, modified spatial cueing designs and electroencephalogram yielded highly inconsistent and unreliable findings, whereas studies employing ‘gaze tracking’ methods (including eye-tracking) provided modest support for a relationship [11]. There may be several explanations why the current study did not replicate previous gaze-tracking findings. One explanation is that the stimuli used here were carefully selected so that participants were presented with ecologically valid photographs of women's bodies that had been validated as underweight and overweight by an independent sample of women [21]. Similar studies have not always used such realistic stimuli (e.g. digitally constructed three-dimensional images of bodies), limiting the generalizability of observed attentional biases and potentially introducing other non-body-related features that attract attention. Therefore, the lack of relationship could reflect the use of more realistic stimuli.

Another possible explanation for the lack of replication of previous gaze-tracking studies may be owing to differences in how body dissatisfaction was measured. A range of scales has been used to measure body dissatisfaction, or body image more broadly [25]. Here, we chose the validated EDI-3 body dissatisfaction subscale to extend the work by Stephen *et al*. [15], which only used a single-item measure; ‘Overall, how satisfied are you with your body’ rated on a scale of 1 (very dissatisfied) to 7 (very satisfied). The EDI-3 subscale focuses on how individuals perceive the size and shape of their bodies. However, the desirability of certain body sizes and shapes has been shown to change over time and differ across ages, cultures and with exposure to Western media [26]. It is possible that this scale did not accurately capture different aspects of body dissatisfaction in our sample. However, an exploratory analysis using the single-item measure ‘I feel satisfied with the shape of my body’ did not change our findings, suggesting that this was not the primary explanation for a lack of replication. Alternatively, the scale may have captured different aspects of body dissatisfaction (e.g. size, shape, tone, attractiveness constructs), but our stimuli were designed to vary based only on the size of the body (lower/higher weight). Therefore, the stimuli may not have motivational relevance for all participants, suggesting the attentional patterns we observed may not be exclusively driven by thin-ideal processes. It was also noted that the relationship between body dissatisfaction and attentional bias was not robust to adjustment for age and BMI. However, body dissatisfaction and BMI are often associated, so it was unclear whether BMI was driving the relationship between body dissatisfaction and attentional bias or whether BMI masked a true relationship owing to collinearity.

A third possible explanation for a lack of replication is that there may be differences in how body dissatisfaction was operationalized. Many previous studies (e.g. [14]) selectively recruited participants with high and low body dissatisfaction scores, which we did not. Our sample had a wide range of body dissatisfaction scores (0–31 out of a maximum of 0–36), and our exploratory analysis comparing the upper and lower quartiles did not change our findings, although it was underpowered. It is therefore possible that an attentional bias may only be present in individuals with more extreme levels of body dissatisfaction. Relatedly, it may be that this relationship only exists in clinical populations with eating disorders. For example, women with eating disorders (e.g. bulimia nervosa and binge eating disorder) have been reported to attend for longer towards, and have poorer disengagement from, low-BMI images of bodies compared to non-clinical control groups [27–29]. However, existing eye-tracking studies in clinical samples are often noted to be of poor quality (e.g. small sample sizes), and very few have assessed the differential relationship between body dissatisfaction and attentional biases in these samples compared to non-clinical controls [30]. This is therefore an important avenue for future research.

Regardless of body dissatisfaction, there was a suggestion of a general attentional bias towards lower-weight bodies. Previous studies have reported both attentional biases towards and away from lower-weight bodies (e.g. [31,32]) suggesting attendance and avoidance. However, the direction of the current findings is similar to Glauert *et al*. [31], a study that used a modified dot probe task and presented lower-weight bodies with higher-weight bodies at the same time. This might suggest that this general bias is observable when both body types are presented, demanding a direct visual comparison, although future studies are needed to understand this further. Our sample of (mostly younger) women was chosen as a population reported to have higher levels of body dissatisfaction and at higher risk of developing eating disorders, depression and other negative health behaviours. It is therefore also possible that this general attentional bias may contribute to higher body dissatisfaction levels and risk of mental health problems in young women, but that this may not be the case in other populations.

The strengths of the current research are that the experimental design and statistical analyses were pre-registered, it was adequately powered based on previous literature, and the stimuli used were ecologically valid. There are also some limitations. First, while we did not restrict the age range of our sample, we largely recruited younger women from the local area, which limits the generalizability of our results to a wider population, e.g. older women, diverse populations and men. Second, our task involves free viewing. While simple and easy to explain to a participant, free-viewing is not a performance-related task and, as such, any participant behaviour is ‘correct’. Such tasks allow a heterogeneous, unconstrained range of idiosyncratic behaviours. This is particularly true for less realistic stimuli and longer viewing durations, as used here. In future studies, an eye-tracking task using more visually realistic and complex stimuli coupled with an overt task may be better for elucidating genuine attentional biases. Third, our exclusion criterion of a current or previous diagnosis of an eating disorder was assessed via self-report, which may not be reliable, and does not account for undiagnosed eating disorder symptoms. Future studies may benefit from using a clinical measure of eating disorder symptomatology.

## 5. Conclusion

In conclusion, our findings did no replicate previous studies showing a relationship between body dissatisfaction and an attentional bias towards lower-weight bodies in women without an eating disorder. This was despite the use of more realistic body stimuli and a validated scale of body dissatisfaction. However, there was weak, but not robust, evidence that all our participants displayed a bias towards lower-weight bodies, which could contribute to higher levels of body dissatisfaction and risk of mental health problems in younger women. Our research highlights the importance of continued efforts to conduct replication studies that are pre-registered and published regardless of null findings. Future studies designed to measure attentional biases in more realistic settings, with visually rich stimuli and tasks, as well as in clinical populations with symptoms of eating disorders, are needed.

## Supplementary Material



## Data Availability

Data are available at the University of Bristol data repository, data.bris [33]. Supplementary material is available online [34].
