# Supplementary material for: No association between body dissatisfaction and attentional bias towards lower-weight bodies in a non-clinical sample of women: an eye-tracking study
Source: R Soc Open Sci. 2026 Oct 28;13(10):252073. doi: 10.1098/rsos.252073 (PMC13714368; doi:10.1098/rsos.252073)
Supplement: Supplementary file 1 [file rsos.252073.zip › rsos.252073/rsos252073_si_001.docx]

**Supplementary Materials**

Supplementary Table 1. Linear regression models of the association between body dissatisfaction and outcome measures after exclusion of three older participants (age outliers).

|  | **Linear regression model 1: unadjusted** |  | **Linear regression model 2: adjusted*** |  |
| --- | --- | --- | --- | --- |
|  | **β [95% CI]** | ***p* value** | **β [95% CI]** | ***p* value** |
| **Fixation duration on lower weight bodies (%)** | -0.239 [-0.560, 0.0832] | 0.14 | -0.0554 [-0.397, 0.286] | 0.75 |
| **Fixation count on lower weight bodies (%)** | -0.158 [-0.446, 0.129] | 0.28 | 0.010 [-0.292, 0.313] | 0.95 |
| **First fixation count on lower weight bodies (%)** | 0.107 [-0.312, 0.527] | 0.61 | 0.210 [-0.253, 0.674] | 0.37 |

Note: percentages are presented relative to trial duration (6 seconds). CI = Confidence Interval

*Adjusted for age and BMI.

Supplementary Table 2. Descriptive statistics of participant characteristics (separated into high and low body dissatisfaction group)

|  | **High BD (N = 16)** | **Low BD (N = 17)** | **Mean Difference [95% CI]** | **p Value** |
| --- | --- | --- | --- | --- |
| **Age** |  |  |  |  |
| Mean (SD) | 28.3 (8.79) | 24.7 (5.11) | 3.60 [-1.61,8.81] | 0.17 |
| Median | 25 | 23 |  |  |
| Range | 18-48 | 19-33 |  |  |
| **BMI** |  |  |  |  |
| Mean (SD) | 26.1 (4.55) | 22.8 (2.63) | 3.29 [0.599,5.98] | 0.02 |
| Median | 25.4 | 23.4 |  |  |
| Range | 19.5-33.3 | 19.1-27.3 |  |  |
| **EDI-3-BD** |  |  |  |  |
| Mean (SD) | 22.8 (4.32) | 6.35 (2.85) | 16.5 [13.8, 19.1] | <0.001 |
| Median | 23 | 6 |  |  |
| Range | 18-31 | 0-10 |  |  |
| **GAD-7** |  |  |  |  |
| Mean (SD) | 5.44 (2.50) | 4.29 (3.06) | 1.14 [-0.837, 3.12] | 0.25 |
| Median | 6 | 4 |  |  |
| Range | 0-9 | 0-12 |  |  |

BD = Body dissatisfaction; CI = Confidence interval; SD = Standard deviation; BMI = Body mass index; EDI-3-BD = Eating Disorder Inventory-3-Body Dissatisfaction subscale (scores range from 0-36); GAD-7 = Generalised Anxiety Disorder-7 (scores range from 0-21)

Supplementary Table 3. Mean (SD) values for fixation data for high BD and low BD groups

|  | **High BD (N = 16)** | **Low BD (N = 17)** | **Mean Difference [95% CI]** | **p-value** |
| --- | --- | --- | --- | --- |
| **Fixation duration (ms)** |  |  |  |  |
| Higher weight body | 2368 (478) | 2112 (529) | 256 [-101, 614] | 0.15 |
| Lower weight body | 2363 (462) | 2637 (579) | -274 [-645, 97.4] | 0.14 |
| **Percentage fixation duration (relative to trial duration) (%)** |  |  |  |  |
| Higher weight body | 38.0 (7.71) | 34.3 (8.67) | 3.72 [-2.10, 9.54] | 0.20 |
| Lower weight body | 38.0 (7.23) | 42.6 (9.24) | -4.67 [-10.5, 1.21] | 0.12 |
| **Fixation count** |  |  |  |  |
| Higher weight body | 8.21 (2.27) | 7.33 (1.69) | 0.883 [-0.555, 2.320] | 0.22 |
| Lower weight body | 8.08 (1.96) | 9.00 (1.95) | -0.917 [-2.31, 0.472] | 0.19 |
| **Percentage fixation count (%)** |  |  |  |  |
| Higher weight body | 40.7 (7.03) | 36.1 (8.36) | 4.52 [-0.958, 10.0] | 0.10 |
| Lower weight body | 40.3 (7.28) | 43.9 (7.58) | -3.61 [-8.89, 1.66] | 0.17 |
| **First fixation count** |  |  |  |  |
| Higher weight body | 9.69 (2.47) | 10.1 (2.28) | -0.371 [-2.06, 1.32] | 0.66 |
| Lower weight body | 10.2 (2.74) | 9.53 (2.55) | 0.658 [-1.23, 2.54] | 0.48 |
| **Percentage first fixation count (%)** |  |  |  |  |
| Higher weight body | 48.9 (12.0) | 51.5 (10.1) | -2.66 [-10.6, 5.28] | 0.50 |
| Lower weight body | 51.1 (12.0) | 48.5 (10.1) | 2.66 [-5.28, 10.6] | 0.50 |

SD = Standard deviation; BD = Body dissatisfaction; CI = Confidence interval; ms = milliseconds

Supplementary Table 4. Linear regression models of the association between body dissatisfaction (low BD group as the reference group) and outcome measures

|  | **Model 1: unadjusted** |  | **Model 2: adjusted*** |  |
| --- | --- | --- | --- | --- |
|  | **β [95% CI]** | **p value** | **β [95% CI]** | **p value** |
| **Primary Outcomes** |  |  |  |  |
| Fixation duration on lower weight bodies (ms) | -274 [-647, 99.6] | 0.15 | -72.8 [-474, 329] | 0.71 |
| Percentage fixation duration on lower weight bodies (relative to trial duration) | -4.67 [-10.6, 1.25] | 0.12 | -0.737 [-6.85, 5.38] | 0.81 |
| **Secondary Outcomes** |  |  |  |  |
| Fixation counts on lower weight bodies | -0.917 [-2.306, 0.472] | 0.19 | -0.161 [-1.66, 1.34] | 0.83 |
| Percentage fixation count on lower weight bodies | -3.61 [-8.90, 1.67] | 0.17 | 0.0328 [-5.32, 5.38] | 0.99 |
| **Exploratory Outcomes** |  |  |  |  |
| First fixation counts on lower weight bodies | 0.658 [-1.22, 2.54] | 0.48 | 1.371 [-0.740, 3.482] | 0.19 |
| Percentage first fixation counts on lower weight bodies | 2.66 [-5.22, 10.5] | 0.50 | 5.00 [-3.98, 14.0] | 0.26 |

BD = Body dissatisfaction; CI = Confidence interval; ms = millieseconds

*Adjusted for age and BMI

Supplementary Table 5. Linear regression models of the association between body dissatisfaction (measured by a single item) and outcome measures

|  | **Model 1: unadjusted** |  | **Model 2: adjusted*** |  |
| --- | --- | --- | --- | --- |
|  | **β [95% CI]** | **p value** | **β [95% CI]** | **p value** |
| **Primary Outcomes** |  |  |  |  |
| Fixation duration on lower weight bodies (ms) | -82.9 [-233, 67.9] | 0.28 | -66.9 [-214, 80.1] | 0.37 |
| Percentage fixation duration on lower weight bodies (relative to trial duration) | -1.58 [-3.99, 0.835] | 0.20 | -1.30 [-3.61, 1.02] | 0.27 |
| **Secondary Outcomes** |  |  |  |  |
| Fixation counts on lower weight bodies | -0.200 [-0.758, 0.358] | 0.48 | -0.111 [-0.657, 0.435] | 0.68 |
| Percentage fixation count on lower weight bodies | -1.15 [-3.31, 1.01] | 0.29 | -0.952 [-3.01, 1.10] | 0.36 |
| **Exploratory Outcomes** |  |  |  |  |
| First fixation counts on lower weight bodies | -0.00293 [-0.806, 0.800] | 0.99 | 0.0534 [-0.772, 0.879] | 0.90 |
| Percentage first fixation counts on lower weight bodies | -0.890 [-4.11, 2.33] | 0.58 | -0.619 [-3.93, 2.69] | 0.71 |

CI = Confidence interval; ms = milliseconds

*Adjusted for age and BMI
